# Patients with hereditary ATTR amyloidosis experience an increasing burden of illness as the disease progresses

**DOI:** 10.1186/1750-1172-10-S1-P58

**Published:** 2015-11-02

**Authors:** David Adams, Oved Amitay, Teresa Coelho

**Affiliations:** 1 Univ Paris-Sud, APHP, Hopital de Bicetre, Service de Neurologie and Centre de Reference des Neuropathies Amyloides Familiales (NNERF), INSERM U1195, Le Kremlin-Bicetre, France; 2 Alnylam Pharmaceuticals, Commercial, 02142, Cambridge, USA; 3 Hospital de Santo Antonio, Unidade Clinica de Paramiloidose, 4099001, Porto, Portugal

## Background

Hereditary ATTR amyloidosis with polyneuropathy, also known as Familial Amyloidotic Polyneuropathy (FAP), is a rare, inherited, progressively debilitating disease with a high unmet medical need. The purpose of this analysis is to assess the impact of this disease on healthcare resource utilization, quality of life, employment status, and activities of daily living (ADLs).

## Methods

A Phase 2 open-label extension study of patisiran in FAP patients was utilized to collect patient-reported outcomes, including EQ-5D, Rasch-built Overall Disability Scale (R-ODS), and a healthcare resource utilization questionnaire.

## Results

The study included 27 patients, 18 males and 9 females, 29-77 years of age. Baseline data are presented for 14 patients with a Polyneuropathy Disability (PND) Score I and 13 patients with a PND Score II or greater. Characterized by FAP Stage, 24 patients are FAP Stage 1 and 3 patients are FAP Stage 2. Two patients (PND Score II or greater) reported a total of six hospitalizations due to FAP in the past 12 months, each for 3 or more nights in duration. Mean EQ-5D scores were 0.82 (PND Score I) and 0.74 (PND Score II or greater). Patients reported their perceived health status on the EQ-VAS with mean scores of 75 (PND Score I) and 60 (PND Score II or greater). Ten patients (8/10 PND Score II or greater) reported they cannot work because of FAP (mean 61 years of age). Patients also reported inability to perform various ADLs. Most commonly, 77% of patients with PND Score II or greater cannot stand for hours (14% in PND Score I) and 69% cannot run (21% in PND Score I).

## Conclusions

FAP patients experience considerable burden of illness early in the course of disease and this burden increases with disease progression. The factors described will be influential in the development of a comprehensive FAP cost-consequence analysis. Additional parameters may also be needed to fully capture the totality of disease burden.

